# Exploring the causal relationship between B lymphocytes and Parkinson’s disease: a bidirectional, two-sample Mendelian randomization study

**DOI:** 10.1038/s41598-024-53287-7

**Published:** 2024-02-02

**Authors:** Jia Song, Yidan Qin, Lin Wang, Wei Quan, Jing Xu, Jia Li, Jiajun Chen

**Affiliations:** Department of Neurology, China-Japan Union Hospital of Jilin University, Changchun, 130033 China

**Keywords:** Parkinson's disease, Risk factors, Parkinson's disease

## Abstract

Parkinson’s disease (PD) is a neurodegenerative disorder with extensive involvement of motor symptoms, imposing a heavy economic burden on patients and society. B lymphocytes, a group of immune cells associated with humoral immunity, have been shown to be involved in the pathogenesis of PD. However, the causal relationship and potential pathogenic effects of B cell in PD remain unclear. Based on the three core hypotheses of the Mendelian randomization (MR) study, we explored causal associations between 190 B-cell immunological traits and 482,730 European individuals (Ncase = 33,674, Ncontrol = 449,056) from genome wide association studies by means of the two-sample bidirectional MR method. The inverse‑variance weighted method was selected as the main approach when conducting MR analysis. Finally, the results were verified by the heterogeneity and horizontal pleiotropy analyses. Five B-cell immunological phenotypes were nominally associated with PD at the significance threshold of *P* < 0.05. Concretely, IgD + CD38− B cell %lymphocyte (OR 1.052, 95% CI 1.001–1.106, *P* = 0.046), CD20 on IgD− CD24− B cell (OR 1.060, 95% CI 1.005–1.117, *P* = 0.032), CD38 on IgD+ CD24− B cell (OR 1.113, 95% CI 1.028–1.206, *P* = 0.009), and BAFF-R on CD20− B cell (OR 1.093, 95% CI 1.010–1.184, *P* = 0.027) were identified as risk factors for PD. Instead, CD38 on Plasma Blast-Plasma Cell (OR 0.894, 95% CI 0.802–0.996, *P* = 0.043) was proved to be protective. However, there is no statistically significant correlation between B cell and PD after Bonferroni correction. The results of reverse MR were negative, avoiding the reverse causal effects. Eventually, the association results were identified as stable across several sensitivity analyses. Briefly, our study might demonstrate the key factor of B cells in PD. Further studies are warranted to clarify the associations for early identification and immunotherapeutic development in PD patients.

## Introduction

Parkinson’s disease (PD) is the second most common neurodegenerative disease, with a global prevalence of more than 6 million individuals in 2016. Recently, the world saw a rapidly growing prevalence especially in China and high-income countries in Europe^1^. PD is classically characterized by multiple motor symptoms, including tremor, bradykinesia, rigidity, and postural instability. The disabling injuries bring crushing burden to the family and the health system worldwide^2^ without effective treatments. Immunotherapy might be a promising area in PD^3^, however, deleting the classification of immune cells is not feasible as it can severely disrupt the immune system. Worse still, the initial clinical diagnosis of PD could be challenging due to the atypicality symptoms^4^. Therefore, the identification of risk factors for PD is of great importance and urgency for the development of diagnostic biomarkers and therapeutic target^5^.

Ample epidemiologic evidence suggests alterations in immune cell populations, especially T cell, could perpetuate the neurodegenerative process of PD^6,7^. However, the role of B cells is less well understood and is being urgently explored in PD^8^. Currently, several clinical studies were designed to explore the counts and phenotypes of B cell in PD with conflicting results^9–14^. Most studies find reduced B cell counts in PD^9,14^. For example, decreased CD19+ B cells were detected in both PD mouse model^15^ and patients^10^. Likewise, five membrane-bound B cell genes (FCRL1, CD19, CD22, CD79A and CD180) were proved to be down-regulated in PD patients^12^. In contrast to previous studies, α-synuclein−/− (KO) mice showed a fourfold reduction on the absolute number of B220+ IgM+ B cells in bone marrow, suggesting the crucial role of α-synuclein in the cytopoiesis of B cell^16^. Another study showed that the percentage of naive B cells was decreased in the blood of PD patients, whereas the percentages of regulatory B cells, plasma blast cells, and double-negative B cells were increased^9^. Given the inconsistent results of the current studies, possibly attributed to the unavoidable shortcomings of observational studies such as confounding factors and selection bias, the complex interaction relationship could not yet be elucidated between B lymphocytes and PD.

Mendelian randomization (MR) analysis is a high statistical efficient method for uncovering causal relationships based on genome wide association studies (GWAS). Superior to observational studies, reverse causation can be avoided in MR, as the mutation of the gene loci precedes the onset of the disease. In addition, the sample size of MR is sufficient to minimize bias. Based on the immune NK cell traits of from a GWAS study^17^, Gong et al. have identified the key role of NK cells in amyotrophic lateral sclerosis through a MR study^18^. However, there are no MR studies on the causal relationship between immune B lymphocytes and PD. Herein, we utilized the MR method to explore the potential role B cells implicated in PD, which offers novel perspectives in the pathogenesis and immune-related therapeutic targets for the disease.

## Materials and methods

### Study design

We assessed the bidirectional causality between 190 B-cell immune traits (Supplementary Data S1) and PD based on a two-sample MR analysis. The genetic variation is adopted as risk factors. Therefore, the selection of valid instrumental variables (IVs) should fulfill three key assumptions: (i) the IVs are strongly associated with exposure; (ii) the IVs are independent of confounders; (iii) the genetic instruments-outcome association is mediated only by the exposures. This study used publicly available data from participant studies that were approved by an ethical standards committee with respect to human experimentation. No separate ethical approval was required in this study.

### GWAS data sources for PD

The GWAS data source for PD, ieu-b-7, was obtained from the International Parkinson’s Disease Genomics Consortium. The GWAS dataset was based on 482,730 European individuals (Ncase = 33,674, Ncontrol = 449,056), and 17,891,936 independent single nucleotide polymorphisms (SNPs) were identified.

### Immunity-wide GWAS data sources

The immune traits of B cell were publicly available from the GWAS Catalog^17^. There were 190 B-cell immunophenotypes altogether, including absolute cell counts (n = 20), median fluorescence intensity (n = 130), and relative cell counts (n = 40). The GWAS analysis was performed according to 3757 European individuals with no overlapping cohorts.

### Selection of IVs

The significance level was set at *P* < 1 × 10^−5^ to select the IVs of immune B-cell trait^17,19^. Afterwards, the linkage disequilibrium in the selected IVs with R^2^ threshold of < 0.001 in the distance of ≥ 10,000 kilobases was clumped^20^. For the inverse MR analysis, the significant threshold was determined as 5 × 10^−8^. The F-statistic was calculated for each IV, and SNPs with F > 10 were retained for subsequent studies.

### MR analysis

After harmonizing the effect alleles across the GWASs from B cell immunophenotypes and PD, we applied the “TwoSampleMR (v.0.5.7)” package in R software (http://www.Rproject.org, v.4.2.2)^21,22^. Then we analyzed by three methods, namely Inverse variance weighted (IVW), MR Egger, and Weighted median. Specifically, IVW was implemented as the main method to examine the overall causal relationship, while MR-Egger and Weighted median for improving the IVW estimates^23^.

### Sensitivity analysis

Cochran’s Q statistic and the corresponding *P*-value were computed to identify heterogeneity. Besides, the MR-Egger intercept was assessed to test horizontal pleiotropy^24^. It is worth stressing that the study need to be redesigned if the calculated* P* value of the MR-Egger intercept was less than 0.05. Meanwhile, the “MR-PRESSO (v.1.0)” package was used to test for the presence of horizontal pleiotropy by removing outlier SNPs^25^. The corresponding forest plot and scatter plot were constructed based on the MR results, and the leave-one-out analysis was performed to evaluate whether the MR estimate was driven or biased by a single SNP^26^.

### Statistics analysis

For a global-level test, a significant two-sided *P*-value was set as 0.05. For region-level analyses, given the 190 MR estimates, a Bonferroni-corrected *P*-value was set as 0.05/190 (2.6 × 10^–4^), and meanwhile *P* < 0.05 was regarded as nominally significant^23^.

## Results

### MR analysis

According to the Bonferroni-corrected *P* value, no immunophenotype had a protective or pathogenic effect on PD. When the threshold was released to *P* < 0.05, five B-cell immunophenotypes had a causal effect on PD as listed: IgD+ CD38− B cell %lymphocyte, CD20 on IgD− CD24− B cell, CD38 on IgD+ CD24− B cell, CD38 on Plasma Blast-Plasma Cell, and BAFF-R on CD20− B cell.

As for the five phenotypes (Supplementary Data S2), 21, 29, 17, 16 and 14 SNPs (Supplementary Data S2) were screened for further genetic association analysis respectively. The F values of all SNPs were greater than 10, suggesting the strong instruments of IVs (Supplementary Data S2). There were no duplications of SNPs among the phenotypes, and no correlation was found between SNPs and outcome (*P* > 1 × 10^–5^).

**Figure 1:**
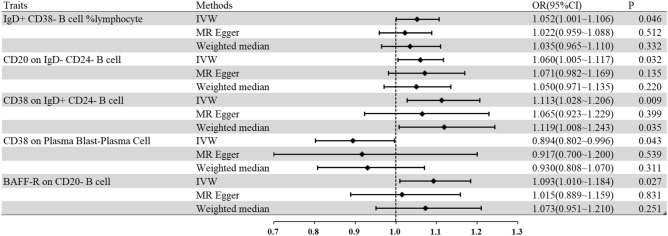
The forest plot about the causal associations between PD and immune B cell traits. *CI* confidence interval, *IVW* inverse variance weighting, *OR* the odds ratio.

The IVW method was estimated for the causal associations between the phenotypic variables and PD (Fig. 1). Concretely, genetically predicted the IgD+ CD38− B cell %lymphocyte demonstrated a nominally causal effect on PD (OR 1.052, 95% CI 1.001–1.106, *P* = 0.046); the CD20 on IgD− CD24− B cell showed a nominally causal effect on PD (OR 1.060, 95% CI 1.005–1.117, *P* = 0.032); the CD38 on IgD+ CD24− B cell exhibited a nominally causal effect on PD (OR 1.113, 95% CI 1.028–1.206, *P* = 0.009); and the BAFF-R on CD20− B cell revealed a nominally causal effect on PD (OR 1.093, 95% CI 1.010–1.184, *P* = 0.027). Apart from the risk factor above, the genetically predicted CD38 on Plasma Blast-Plasma Cell was associated with a lower risk of PD (OR 0.894, 95% CI 0.802–0.996, *P* = 0.043). When the reverse MR analysis was performed, the MR results were all negative (*P* > 0.05, Supplementary Data S3).

### Sensitivity analysis

Cochran’s Q statistic was calculated using MR Egger and IVW method, and the results showed that selected IVs had no heterogeneity (*P* > 0.05, Supplementary Data S4). The *P* value of the MR-PRESSO and MR Egger intercept were greater than 0.05 (Supplementary Data S5), suggesting that there was no horizontal pleiotropy, and the related scatter plot was shown in Supplementary Fig. S1. What’s more, there were no outlier SNPs regarding to the leave-one-out results (Supplementary Fig. S2).

## Discussion

PD is a progressive neurodegenerative disease that affects multi-systems, leading to a severe decrease in the quality of patients’ life. Neuroinflammation and immune system dysfunction have been implicated in the development of motor and non-motor symptoms, which can precede the diagnosis by decades^8^. Emerging supportive evidence is as follows for the involvement of the immune system in PD: (1) PD and the autoimmune diseases, such as Crohn’s disease, share a common genetic pathway^27,28^; (2) Patients with PD were detected with antibodies including anti-α-syn^29,30^, to which the nigrostriatal dopamine neurons were immunoreactive, and the IgG receptors were expressed on the activated microglia nearby^31^; (3) PD is accompanied by alterations in oral and gut microbiota with localized inflammatory infiltration^32,33^; (4) PD models could benefit from the antibiotic treatment^34,35^. Thereinto, immune cells are important components including B cells, T cells and NK cells. Here, we innovatively harnessed MR to validate the association between immune B cells and PD. A total of five B cell subtypes had a nominally correlation with the development of PD, among which IgD+ CD38− B cell %lymphocyte, CD20 on IgD− CD24− B cell, CD38 on IgD+ CD24− B cell, BAFF-R on CD20− B cell are the causative factors of PD, while CD38 on Plasma Blast-Plasma Cell is protective against PD. Finally, we performed sensitivity analyses to access the robustness of the MR results.

The innate immunity and adaptive immunity are cooperatively engaged in the neuroimmune crosstalk of PD. In this study, we targeted the immune B cells of adaptive immunity, which is a crucial component of humoral immunity and closely related to the production of antibody. B cells can be categorized into different subtypes according to the immunomarkers^36^. At present, the complex immune-brain interaction between B cells and PD is still being explored, which is essential for the in-depth exploration of PD pathologic process. On the one hand, research on antibody against PD contributes to the identification of diagnostic and therapeutic biomarkers. Yanamandra et al. found higher antibody levels towards monomeric a-synuclein in the blood sera of PD patients compared to controls with the responses decreased in PD progression^37^, suggesting a complex association of B-cell-related humoral immunity with the PD course. For the production of α-syn antibodies in vivo, different studies have found that the cross reactivity between herpes simplex virus 1^38^, Helicobacter pylori^39^ and human α-synuclein peptides, revealing the participation of environmental factors in the immunization process. In addition to α-syn antibody, anti-melanin humoral response was present in PD, which was more active in the early stages^40^. On the other hand, scholars further explored the specific subtypes of PD and B cells with the continuous development of flow cytometry technology. Kirsten’s team found fewer B cells in PD patients especially at higher risk for early dementia; and patients got better motor scores with a higher proportion of regulatory B cells, implying the potential protective effect on PD^11^. In addition, transcriptome analysis and quantitative-RT-PCR in female patients were performed to reveal the decreased expression in uniquely B cell-expressed IGHM and IGHD, the B cell surface molecules CD19, CD22 and CD79A, and the B cell gene regulator, PAX5^13^.

In this study, some B-cell phenotypes previously unreported were identified with potential causal effects on PD, which might be related to the bias in observational studies and the insufficient discrimination of B-cell immune traits.

Bm1 (IgD+ CD38−) mainly represents virgin naive cells, which implicates that B-cell-derived processes might be involved in PD lesions according to the study. Despite the limited supporting literature, IgD+ CD38− B cell %lymphocyte might be crucial in PD pathogenesis, which need further study.

CD20 is a B cell-specific membrane protein implicated in the resting state of B cells, resulting in the usage of anti-CD20 antibodies for B cell depletion therapies^41^. In terms of the nervous system, B1 cells, with the biomarkers such as CD20+, are a group of B cells secreting natural autoantibodies, which could recognize neural self-antigens^42^. Recently, CD20 monoclonal antibodies were prominent in ameliorating the progression of multiple sclerosis, making it possible for α-synuclein autoantibodies in PD passive immunization strategies^43^. Therefore, the elevation of CD20 on IgD− CD24− B cell might be related to the production of autoantibodies in PD.

Interestingly, the causal effects on PD of CD38 were contradictory in the IgD+ CD24− B cell and Plasma Blast-Plasma Cell according to the MR results. CD38 was proved critical in pathological process by modulating homeostasis, inflammation, NAD^+^-related metabolism and autoimmune responses^44^. On the one hand, IgD is crucial for the transition from a high degree of primary autoreactivity to a secondary antigen-specific antibody response^45^. CD24 immunologically discriminates between damage-associated and pathogen-associated molecular patterns derived from pathogens, which could suppress the hyperinflammatory response^46^. Therefore, IgD+ CD24− B cell might play a role in the activation of inflammation, accounting for the potential casual effect in PD inflammatory process. On the other hand, CD38 is mainly regarded as the biomarker of the Plasma Blast-Plasma cell, particularly highly and uniformly expressed in Multiple Myeloma, a cancer characterized by proliferation of malignant plasma cells in the bone marrow. Notably, CD38 is essential in the escape of tumor cells from the control of the immune system, making it a target for novel therapeutic strategies^47^. Accordingly, the CD38 Plasma Blast-Plasma cell might act as an immunosuppressant in PD, a disease with abnormal immune activation, to demonstrated the protective effect. Similarly, another MR study suggested causal roles of several proteins, such as CD38, GPNMB, and ADAM15 in PD based on the protein quantitative trait loci in the cerebrospinal fluid^48^. Given the results in different studies, more research on the role of CD38 in PD will be valuable for uncovering the underlying pathogenesis and developing the immunotherapy for the disease.

As for BAFF-R, Zhang et al. found elevated serum levels of BAFF and its negatively correlated with the Unified Parkinson’s Disease Rating Scale (UPDRS) III score in PD, in line with our results about BAFF-R^9^. Existing research proved that the overexpression of BAFF could lead to the expansion of activated B cells, particularly in hypergammaglobulinemia, autoantibody production and immune complex deposition^49^. Briefly, the overexpression of BAFF-R might be involved in the process of autoantibody production of PD similar to CD20. To sum up, alterations in B-cell immune markers represent the activated immune responses in the course of PD.

There was no significant correlation between B-cell phenotypes and PD when adopting the Bonferroni-corrected *P*-value. When IVW was screened according to *P* < 0.05, we yielded 5 positive results, and the OR values of the other two methods were directional consistency^50^, proving the accuracy of the results. Generally speaking, B cells are broadly involved in the pathogenesis of PD through humoral immunization.

In this paper, a comprehensive MR analysis was utilized for the first time to figure out the relationship between B cells and PD, providing GWAS proofs for further clinical studies. The strengths of this study are listed: firstly, the reverse causality was avoided, which facilitates the elaboration on the sequence of B-cell abnormality and PD; secondly, the population bias could be avoided as the large sample size of GWAS; last but not least, this study is of great clinical significance as a theoretical basis for the identification of diagnostic biomarkers and the development of immunotherapeutic targets.

However, there are some limitations in our study: (1) ieu-b-7 is assessed from the International Parkinson’s Disease Genomics Consortium with no overlapping cohorts. However, the study was conducted merely in a European population without the refinement of the sample structure and the subgroup analysis matched by age, sex, original disease, etc., which might lead to the demographic bias of the MR results. Future studies could be designed among other ethnicities and stratified by gender and other demographic characteristics; (2) the outcome of PD could be further categorized into different subtypes (e.g., PD with dementia); and (3) it could be verified in PD patients and basic researches for applying in clinic and uncovering the underlying mechanisms.

## Conclusion

In conclusion, the MR analysis presented the possible genetic association between B lymphocytes and PD. The intervention against the corresponding B-cell immune molecule provides a target for PD immunotherapy, and the immunophenotypes of B lymphocytes are promising diagnostic biomarkers deserving further investigation.

### Supplementary Information

Supplementary Information 1.

Supplementary Information 2.

Supplementary Information 3.

Supplementary Information 4.

Supplementary Information 5.

Supplementary Figure S1.

Supplementary Figure S2.

Supplementary Legends.

## Data Availability

All data used in the study were obtained from published articles or publicly available GWAS platform (https://gwas.mrcieu.ac.uk/), and all data can be obtained for free.
