# Supplementary figures and images for: Exploring the causal relationship between B lymphocytes and Parkinson’s disease: a bidirectional, two-sample Mendelian randomization study
Source: Sci Rep. 2024 Feb 2;14:2783. doi: 10.1038/s41598-024-53287-7 (PMC10837417; doi:10.1038/s41598-024-53287-7)

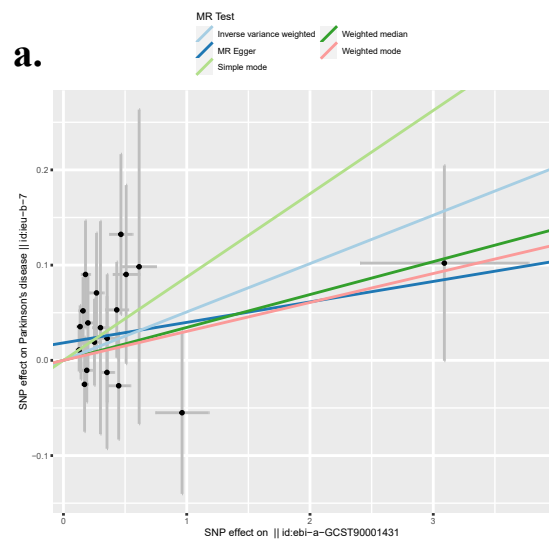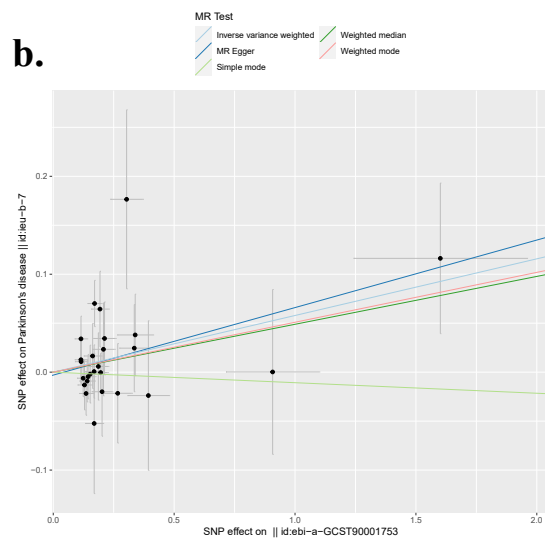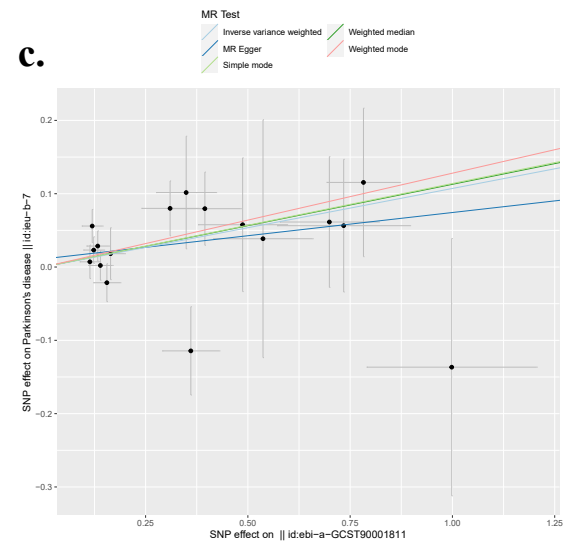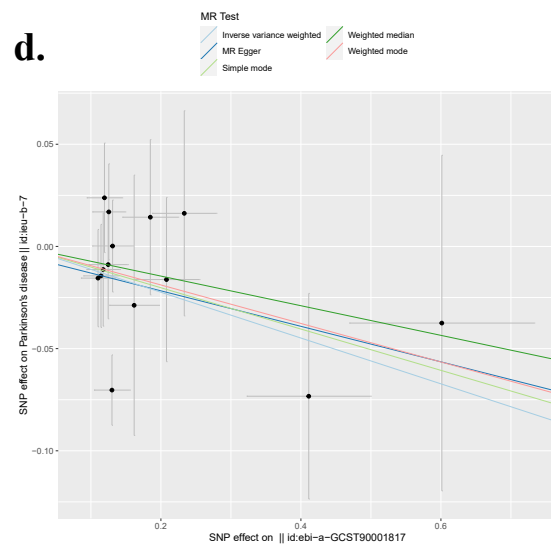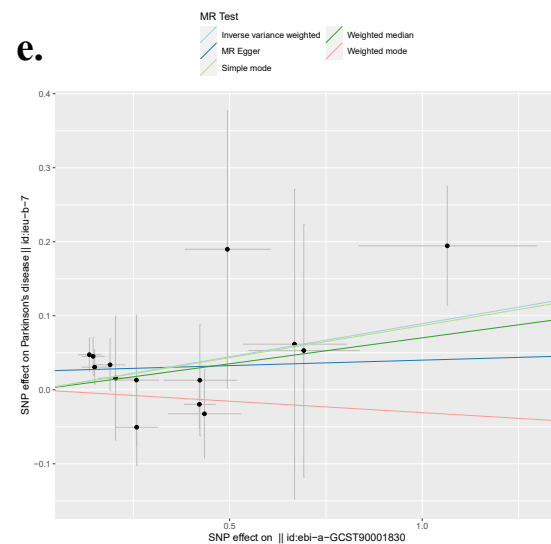

Supplement: Supplementary file 6 — Supplementary Figure S1. [file 41598_2024_53287_MOESM6_ESM.pdf]

**a.**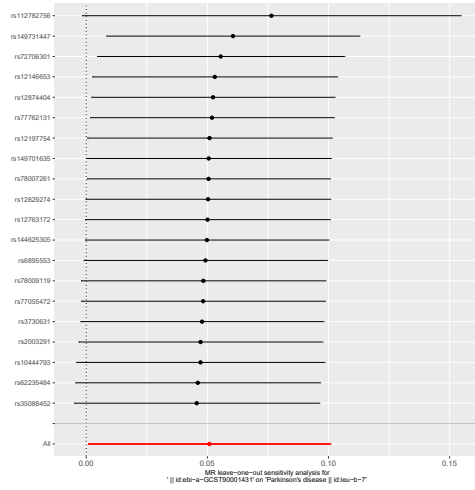**b.**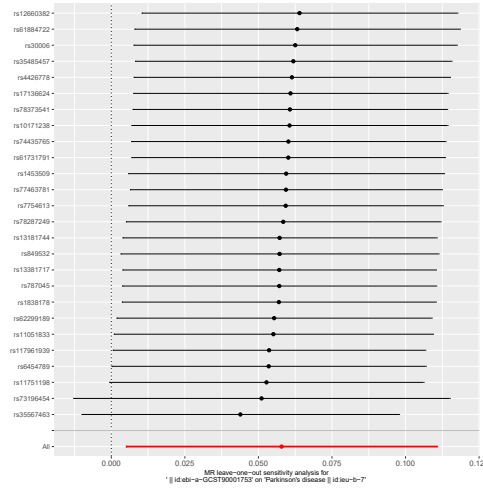**c.**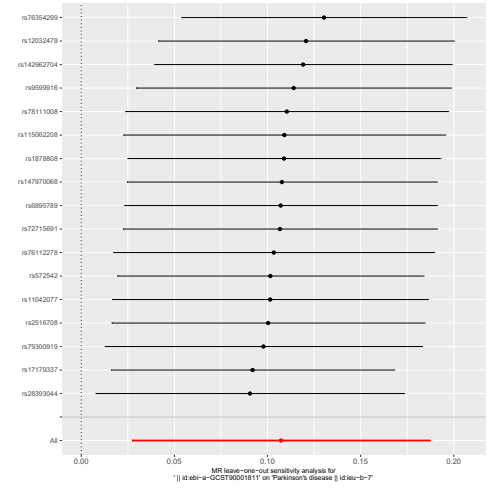**d.**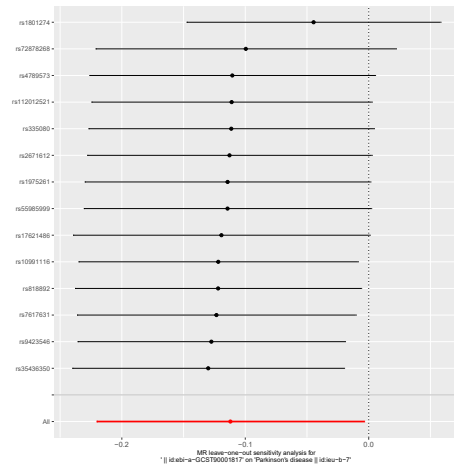**e.**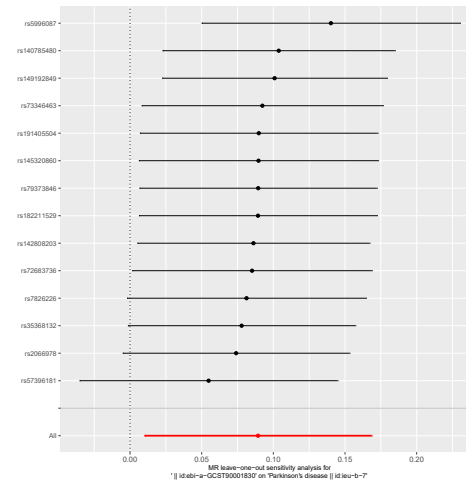

Supplement: Supplementary file 7 — Supplementary Figure S2. [file 41598_2024_53287_MOESM7_ESM.pdf]
