# Supplementary material for: Exploring the causal relationship between B lymphocytes and Parkinson’s disease: a bidirectional, two-sample Mendelian randomization study
Source: Sci Rep. 2024 Feb 2;14:2783. doi: 10.1038/s41598-024-53287-7 (PMC10837417; doi:10.1038/s41598-024-53287-7)
Supplement: Supplementary file 8 — Supplementary Legends. [file 41598_2024_53287_MOESM8_ESM.docx]

**Exploring the causal relationship between B Lymphocytes and Parkinson’s disease: a bidirectional, two-sample Mendelian randomization study**

Supplementary Data S1: The details of the 190 B-cell immune traits.

Supplementary Data S2: The SNPs information for the 5 positive immunological traits.

Supplementary Data S3: The reverse MR results of the study.

Supplementary Data S4: The Cochran's Q statistic results of the heterogeneity analysis.

Supplementary Data S5: The MR-PRESSO (sheet 1) and MR Egger intercept (sheet 2) results of the horizontal pleiotropy analysis.

Supplementary Figure S1: The scatter plots of the MR analysis.

Supplementary Figure S2: The leave-one-out plots of the MR analysis.
